# Does the Relationship between Age and Brain Structure Differ in Youth with Conduct Disorder?

**DOI:** 10.1007/s10802-024-01178-w

**Published:** 2024-04-01

**Authors:** Sarah Koerner, Marlene Staginnus, Harriet Cornwell, Areti Smaragdi, Karen González-Madruga, Ruth Pauli, Jack C. Rogers, Yidian Gao, Sally Chester, Sophie Townend, Anka Bernhard, Anne Martinelli, Gregor Kohls, Nora Maria Raschle, Kerstin Konrad, Christina Stadler, Christine M. Freitag, Stephane A. De Brito, Graeme Fairchild

**Affiliations:** 1 Department of Psychology, University of Bath, Bath, UK; 2 Child Development Institute, Toronto, Canada; 3 Department of Psychology, Middlesex University, London, UK; 4 Centre for Human Brain Health, School of Psychology, University of Birmingham, Birmingham, UK; 5 Institute for Mental Health, School of Psychology, University of Birmingham, Birmingham, UK; 6 Department of Child and Adolescent Psychiatry, Psychosomatics and Psychotherapy, University Hospital Frankfurt, Goethe University, Frankfurt Am Main, Germany; 7 School of Psychology, Fresenius University of Applied Sciences, Frankfurt, Germany; 8 Child Neuropsychology Section, Department of Child and Adolescent Psychiatry, Psychosomatics and Psychotherapy, University Hospital, RWTH Aachen, Aachen, Germany; 9 Department of Child and Adolescent Psychiatry, Medical Faculty, TU Dresden, Dresden, Germany; 10 Department of Child and Adolescent Psychiatry, Psychiatric University Hospital, University of Basel, Basel, Switzerland; 11 Jacobs Center for Productive Youth Development at the University of Zurich, Zurich, Switzerland; 12 Neuroscience Centre Zurich (ZNZ), University and ETH Zurich, Zurich, Switzerland; 13 JARA- Brain Institute II, Molecular Neuroscience and Neuroimaging, RWTH Aachen and Research Centre Juelich, Juelich, Germany

**Keywords:** Conduct disorder, Antisocial behaviour, Brain development, Surface-based morphometry, Cortical thickness, Surface area

## Abstract

Conduct disorder (CD) is characterised by persistent antisocial and aggressive behaviour and typically emerges in childhood or adolescence. Although several authors have proposed that CD is a neurodevelopmental disorder, very little evidence is available about brain development in this condition. Structural brain alterations have been observed in CD, and some indirect evidence for delayed brain maturation has been reported. However, no detailed analysis of age-related changes in brain structure in youth with CD has been conducted. Using cross-sectional MRI data, this study aimed to explore differences in brain maturation in youth with CD versus healthy controls to provide further understanding of the neurodevelopmental processes underlying CD. 291 CD cases (153 males) and 379 healthy controls (160 males) aged 9–18 years (M_age_ = 14.4) were selected from the European multisite FemNAT-CD study. Structural MRI scans were analysed using surface-based morphometry followed by application of the ENIGMA quality control protocols. An atlas-based approach was used to investigate group differences and test for group-by-age and group-by-age-by-sex interactions in cortical thickness, surface area and subcortical volumes. Relative to healthy controls, the CD group showed lower surface area across frontal, temporal and parietal regions as well as lower total surface area. No significant group-by-age or group-by-age-by-sex interactions were observed on any brain structure measure. These findings suggest that CD is associated with lower surface area across multiple cortical regions, but do not support the idea that CD is associated with delayed brain maturation, at least within the age bracket considered here.

**Supplementary Information:**

The online version contains supplementary material available at 10.1007/s10802-024-01178-w.

## Introduction

Conduct disorder (CD) is a psychiatric disorder that usually emerges in childhood or adolescence. It is characterised by persistent antisocial behaviour that violates others’ rights or societal norms such as theft, physical violence towards people or animals, or vandalism (American Psychiatric Association [APA], 2013). The prevalence of CD is estimated at approximately 3–4% for boys and 1–2% for girls, and it is one of the leading reasons for referral to child mental health services in the UK and the USA (Fairchild et al., 2019).

CD is related to poorer physical health, educational problems, and an increased risk of mental and substance use disorders (Erskine et al., 2016; Rivenbark et al., 2018). Furthermore, while CD can be limited to childhood and/or adolescence, it can also persist across the lifespan and lead to the development of antisocial personality disorder and criminal behaviour in adulthood, thereby causing a high individual, economic and societal burden (Fairchild et al., 2019; Scott et al., 2001). Despite an increase in research on CD in recent years, it remains largely under-recognised and under-treated by mental health services (Coghill, 2013). Hence, developing new prevention and intervention models is imperative, meaning that research into the underlying causes and development of CD is crucial.

Neurodevelopmental theories of CD suggest that the condition is related to dysfunction in cortical and subcortical regions of the brain, thereby leading to neurocognitive deficits (Blair et al., 2014). Previous studies have identified impairments in decision making (Fairchild et al., 2009), emotion recognition, learning and regulation (Kohls et al., 2020; Raschle et al., 2019), as well as responses to fear-inducing stimuli (Fanti et al., 2016) and affective empathy (Martin-Key et al., 2020) in CD. Structural MRI studies have demonstrated alterations in cortical and subcortical brain regions associated with these neurocognitive functions in youth with CD when compared to healthy controls (HCs). For instance, two meta-analyses employing voxel-based morphometry (VBM) reported lower gray matter volume (GMV) in several brain regions including the insula, left fusiform gyrus, medial and superior frontal gyrus, as well as the amygdala, in the CD group (Noordermeer et al., 2016; Rogers & De Brito, 2016).

Other investigations exploring brain structural changes in individuals with CD have utilized surface-based morphometry (SBM). Unlike VBM, which estimates gray matter volume and density throughout the brain, SBM distinguishes between two aspects of cortical structure: cortical thickness (CT) and surface area (SA). Since CT and SA may have different developmental trajectories and genetic underpinnings (Panizzon et al., 2009), separate investigation of CT and SA provides valuable insights into the specific cortical changes associated with CD, enhancing spatial resolution and anatomical precision (Raznahan et al., 2011).

Lower CT has been identified in regions such as the ventromedial prefrontal cortex (vmPFC)/orbitofrontal cortex (OFC), fusiform and precentral gyrus, superior temporal cortex and precuneus in youth with CD (e.g., Fairchild et al., 2015; Smaragdi et al., 2017; Wallace et al., 2014). Additionally, reductions in SA were found in areas such as the precentral and inferior temporal cortex as well as the OFC (e.g., Fairchild et al., 2015; Jiang et al., 2015). These structural alterations have been linked to neurological functions such as emotion regulation, affective empathy and reward processing (Fairchild et al., 2015; Kohls et al., 2020). Overall, findings suggest that CD may be related to structural differences in cortical and subcortical regions that are implicated in neurocognitive processes that are impaired in youth with this condition.

While existing cross-sectional research has provided indirect support for the neurodevelopmental theory of CD, findings across the literature are still inconsistent. For example, reduced amygdala volume has been repeatedly reported in CD (Fairchild et al., 2011, 2013; Huebner et al., 2008; Sterzer et al., 2007) while other studies observed no significant group differences in this region (Dalwani et al., 2011; De Brito et al., 2009) – although it should be noted that the latter studies focused on youth with conduct problems rather than CD.

These replicability issues could be due to methodological differences between studies, small sample sizes, or a failure to control for influential factors such as sex (Smaragdi et al., 2017), callous-unemotional traits (Wallace et al., 2014), age-of-onset subtype (Jiang et al., 2015), and comorbid disorders such as attention-deficit/hyperactivity disorder (ADHD; Noordermeer et al., 2016). Additionally, heterogeneity of findings across studies may also be linked to differences in the age ranges that were included (e.g., Fairchild et al., 2015 [16–21 years]; Wallace et al., 2014 [10–18 years]; De Brito et al., 2009 [11–13 years]).

Neuroimaging studies of brain maturation in typically developing children suggest that CT, SA, as well as cortical and subcortical volumes follow both linear and non-linear patterns that depend on the specific brain region and parameter under investigation. Bethlehem et al. (2022) found CT to reach its peak around 1.7 years, followed by GMV at around 5.9 years and SA between 11–12 years, with regional variation (primary sensory regions maturing before those responsible for higher-cognitive functions), as well as sex differences (earlier brain maturation in females).

Regarding subcortical volumes, the caudate, putamen, globus pallidus, and nucleus accumbens reach their peaks during the first decade of life and show a gradual decrease thereafter. In contrast, developmental trajectories of the amygdala, thalamus, and hippocampus follow a more flattened inverted U-curve, with their peak volumes occurring later during the second and third decades of life, remaining relatively stable thereafter (Dima et al., 2022).

Given what is known about the brain’s protracted development, some studies of childhood psychiatric disorders have suggested that differences between clinical groups and HCs are not characterised by stable structural differences, but rather by deviations in maturational trajectories. For instance, Shaw and colleagues (2007) investigated cortical maturation in children with ADHD and showed that this group reached peak CT values approximately 3 years after their typically-developing peers (10.5 versus 7.5 years). Hence, it was proposed that ADHD is characterised by delayed brain maturation rather than stable differences in brain structure across the lifespan. Similarly, a large longitudinal MRI study investigating brain maturation in autism spectrum disorder (ASD) reported abnormally accelerated brain growth in early childhood (2–4 years) in this population. Subsequently, brain maturation appeared to slow down and was followed by accelerated decreases in total brain volume in the ASD group compared to HCs (Courchesne et al., 2011). These studies provide evidence for abnormal brain maturation in youth with neurodevelopmental disorders. In addition, they suggest that structural differences between cases and controls might be age-specific, which could result in inconsistent findings when investigating group averages in samples of varying age ranges, as has been the case in the CD literature.

Based on the previously described evidence of brain structural alterations in CD as well as findings of abnormal brain maturation in other neurodevelopmental disorders, it has been suggested that CD might also be associated with delays in brain development. De Brito and colleagues (2009) addressed this by comparing cross-sectional sMRI data of 11- to 13-year-old HCs to boys high in conduct problems and callous-unemotional traits (CP + CU). HCs displayed a decrease in gray matter concentration in the medial orbitofrontal and dorsal anterior cingulate cortex, whereas boys with CP+CU displayed little change or even an increase in gray matter concentration with age. Furthermore, Oostermeijer et al. (2016) performed a longitudinal study investigating the relationship between conduct problems and brain structure trajectories in adolescents (who were assessed at ages 12, 16 and 19). The sample was grouped based on conduct problems trajectories (stable low, intermediate and desisting), and analyses revealed slower cortical thinning in the desisting (i.e., high and then declining) CPs group in the anterior cingulate and dorsolateral prefrontal cortices. The desisting group also exhibited a sustained increase in hippocampal volume over time, whereas the intermediate group displayed minimal changes, and the stable low CP group demonstrated a comparatively modest and gradual increase. A recent longitudinal study by Albaugh and colleagues (2023) investigated the relationship between conduct problems and cortical thinning in a large sample (N = 1039) that were scanned twice over a period of 5 years from late adolescence. Using vertex-based models they found an association between higher levels of CP and accelerated cortical thinning in the bilateral insula and inferior parietal cortices, and the left inferior frontal gyrus, and rostral anterior and posterior cingulate cortices. These studies provide evidence for altered brain maturation in youth with conduct problems, however, they used non-clinical samples. Therefore, in order to gain further insight into the neurodevelopmental processes underlying CD, all participants in the current study underwent a comprehensive psychiatric assessment to confirm research diagnoses of CD in the clinical group, as well as assess for other common comorbid conditions. Additionally, we performed group-by-age (and group-by-age-by-sex) interaction analyses in a sample including a greater number of youth with CD than most previous analyses and further differentiated between different cortical structure parameters to provide a more comprehensive characterisation of cortical structure alterations.

Building on existing research, the current study aimed to enhance our understanding of neurodevelopmental processes in CD by investigating how brain development in youth with diagnoses of CD compares to that observed in HCs. The sample was selected from the largest cross-sectional structural MRI dataset of youth with CD available to date, and data from youth with CD and HCs were analysed to test for group differences in CT, SA and subcortical volumes and interactions between age and group as well as group-by-age-by-sex interactions. We aimed to replicate previous findings of lower CT or SA in frontal regions and lower volume of subcortical regions such as the amygdala in the CD group compared to HCs. Additionally, based on previous research with other childhood psychiatric disorders such as ADHD, we expected brain maturation to be delayed in CD compared to HC participants, particularly in frontal, temporal and limbic regions.

## Methods

### Participants

The sample used in this study included 291 participants with CD (153 males) and 379 healthy controls (160 males) aged 9–18 years (see Table 1). The sample was selected from the FemNAT-CD study (Freitag et al., 2018), where participants were recruited from schools, mental health clinics, youth offending services, community outreach and youth welfare institutions. The FemNAT-CD study was conducted at various sites across Europe (see Table S1 In Supplement), and ethical approval and informed consent was obtained locally at each site. Ethical approval for the current study was obtained from the University of Bath Psychology Research Ethics Committee (Ethics reference: 19–297).

**Table 1:** Demographic and clinical characteristics of the sample

**Characteristic/ Variable**	**Female CD (n = 138)**	**Female HC** **(n = 219)**	**Male CD** **(n = 153)**	**Male HC** **(n = 160)**	***T*group *(p)***	***T*sex *(p)***	***F*group x sex *(p)***
**Age (years), mean (SD)**	14.77 (2.01)	14.38 (2.54)	14.11 (2.50)	14.15 (2.57)	0.75 (0.455)	2.10 (**0.036**)	1.26 (0.263)
**Estimated IQ, mean (SD)**	96.05 (12.99)	103.27 (11.65)	94.63 (11.96)	104.28 (11.34)	-8.93 **(< 0.001**)	0.86 (0.388)	1.78 (0.183)
**CD symptoms, mean (SD)**	5.09 (2.54)	0.06 (0.26)	5.25 (2.36)	0.14 (0.69)	30.41 **(< 0.001**)	-2.89 (**0.004**)	0.08 (0.775)
**Total ICU, mean (SD)**	32.58 (11.26)	15.98 (7.35)	35.38 (12.41)	18.56 (6.98)	16.69 **(< 0.001**)	-4.60 **(< 0.001**)	0.16 (0.900)
**Lifetime ADHD diagnosis, n (%)**	48 (34)	0 (0)	67 (44)	0 (0)	X^2^ = 201.91 **(< 0.001)**	X^2^ = 7.76 (**0.005**)	X^2^ = 0.00 (0.992)
**Missing**	19 (14)	10 (5)	19 (12)	8 (5)			
**Lifetime ODD diagnosis, n (%)**	106 (77)	1 (1)	110 (72)	1 (1)	X^2^ = 406.47 **(< 0.001)**	X^2^ = 2.80 (0.094)	X^2^ = 4.10 **(0.043)**
**Missing**	1 (1)	1 (1)	5 (3)	6 (4)			
**Lifetime Depression diagnosis, n (%)**	55 (40)	6 (3)	27 (18)	0 (0)	X^2^ = 100.62 **(< 0.001)**	X^2^ = 9.99 **(0.002)**	X^2^ = 14.98 **(< 0.001)**
**Missing**	1 (1)	8 (4)	5 (3)	6 (4)			
**Lifetime Anxiety diagnosis, n (%)**	36 (26)	4 (2)	24 (16)	4 (3)	X^2^ = 60.78 **(< 0.001)**	X^2^ = 0.80 (0.370)	X^2^ = 9.91 **(0.002)**
**Missing**	1 (1)	8 (4)	5 (3)	6 (4)			
**Pubertal development, n (%)**					X^2^ = 8.28 (0.076)	X^2^ = 100.79 (**< 0.001**)	X^2^ = 9.61 **(0.047)**
**Prepubertal**	2(2)	7 (3)	13 (8)	14 (9)			
**Early pubertal**	1 (1)	8 (4)	20 (13)	23 (14)			
**Mid-pubertal**	7 (6)	33 (15)	31 (20)	46 (28)			
**Late pubertal**	81 (59)	101 (46)	49 (32)	59 (37)			
**Post-pubertal**	20 (14)	53 (25)	7 (5)	6 (4)			
**Missing**	27 (20)	17 (8)	33 (22)	12 (7)			
**Handedness, n (%)**					X^2^ = 4.17 (0.124)	X^2^ = 0.05 (0.975)	X^2^ = 9.38 **(0.009)**
**Right**	98 (71)	178 (81)	109 (71)	141 (88)			
**Left**	12 (9)	27 (12)	24 (16)	10 (6)			
**Ambidextrous**	4 (3)	1 (1)	2 (1)	3 (2)			
**Missing**	24 (17)	13 (6)	18 (12)	6 (4)			

*CD* Conduct Disorder, *HC* healthy control, *ADHD* attention-deficit/hyperactivity disorder, *ICU* Inventory of Callous-Unemotional traits, *ODD* Oppositional defiant disorder

### Measures

CD and comorbid externalising and internalising disorders such as ADHD, oppositional defiant disorder (ODD), and Major Depressive Disorder were assessed based on a diagnostic interview with the participants and their parents or carers, the Schedule for Affective Disorders and Schizophrenia for School-Age Children-Present and Lifetime version (K-SADS-PL; Kaufman et al., 1997). Diagnostic interviews were administered by trained staff, and high interrater reliability was established (Cohen’s kappas = 0.84 – 1.00). The parent-report version of the Inventory of Callous-Unemotional traits (ICU; Frick, 2004) was used to assess callous-unemotional (CU) traits. IQ was estimated using the matrix reasoning and vocabulary sub-tests of the Wechsler Abbreviated Scale of Intelligence (Wechsler, 1999) at UK sites. German and Swiss sites used the vocabulary and matrix reasoning subtests of the Wechsler Scales for Children (WISC-III-R/IV) (Wechsler, 1991, 2003) for those aged 9–16 and the adult version (WAIS-III/IV) (Wechsler, 1997, 2008) for those aged ≥ 17 years. The self-report Pubertal Development Scale (Petersen et al., 1988) was used to assess pubertal development. Exclusion criteria included IQ < 70 as well as a history of head trauma, psychosis, autism spectrum disorders and standard sMRI exclusion criteria (e.g., metal braces). HCs were free of any current psychiatric or neurological disorders and past CD, ODD or ADHD, as assessed by the K-SADS-PL.

### Image Acquisition and Processing

MRI data were collected using Siemens 3 T or Philips 3 T scanners and T1-MPRAGE scans were obtained (see Table S3 for more details). Structural MRI image quality was visually inspected and excluded in the case of severe movement, artefacts or evidence of pathology. Subsequently, the T1-weighted images were processed using FreeSurfer v5.3.0, which involved skull stripping and the segmentation of white and grey matter in order to create the pial and white surface as well as the parcellation of cortical and subcortical structures (see Fischl, 2012, for more details). CT and SA were estimated at each vertex and subcortical volumes were calculated based on FreeSurfer’s volume-based stream. Mean CT and total SA values were extracted for all cortical regions as defined by the Desikan-Killiany atlas (Desikan et al., 2006), resulting in CT and SA values for 34 bilateral hemisphere regions. Likewise, volume for seven subcortical regions (amygdala, caudate, hippocampus, nucleus accumbens, pallidum, putamen and thalamus) was obtained per hemisphere based on FreeSurfer’s aseg atlas. Average CT and total SA per hemisphere and total intracranial volume were also computed. Following the protocols of the Enhancing Neuro-Imaging and Genetics through Meta-Analysis (ENIGMA) consortium (http://enigma.usc.edu/protocols/imaging-protocols/), stringent region-by-region quality control procedures were performed blind to group status. This included visual inspection of all parcellations and segmentations, statistical assessment for outliers and inspection of histograms for distributional irregularities. Combining information across the ENIGMA quality control assessments, just 2.4% of regions in the current sample were failed.

### Statistical Analysis

Demographic and clinical group and sex differences were determined using independent samples *t*-tests or Chi-square tests, while group-by-sex interactions were computed using univariate analyses of variance and Chi-square tests. These analyses were conducted using IBM SPSS Statistics 27, while all neuroimaging analyses were performed in R (v4.1.1) using the extracted values from FreeSurfer. For the analysis of brain structure, the values for the left and right hemispheres were combined by computing averages of the CT, SA and subcortical measures. Firstly, group and sex were dummy-coded and group, age and sex were mean-centred. Subsequently, we used linear regression analyses to test for main effects of group (CD versus HC), main effects of age, group-by-age interactions and group-by-age-by-sex interactions. Analyses also included all other possible two-way interactions (group-by-sex & age-by-sex). CT and SA were analysed separately for each cortical region. We controlled for site and sex across all analyses, and ICV additionally in the SA and subcortical volume analyses, to account for individual differences in brain size. Assumptions of linearity, normality, homoscedasticity and multicollinearity were checked, and no violations were detected. Multiple comparisons across brain regions and morphometric parameters were controlled for by using the False Discovery Rate approach (FDR; Benjamini & Hochberg, 1995) and adjusted *p*-values were reported. Effect sizes were determined using Cohen’s *d*, which are commonly interpreted as small (d = 0.2), medium (d = 0.5) and large (d = 0.8) (Cohen, 1988). Sensitivity analyses were conducted to control for IQ and lifetime ADHD (a categorical variable), as CD is strongly associated with lower IQ as well as comorbid ADHD (Anney et al., 2008; Deater-Deckard et al., 2009). Additionally, we controlled for lifetime diagnoses of depression or anxiety disorders to account for the presence of internalising comorbidity. It was not possible to control for ODD because the majority (72%) of the CD group had comorbid ODD which aligns with established comorbidity rates of 96% in clinical samples and 60% in community samples (Rowe et al., 2010).

The impact of CU traits on the findings was estimated by repeating the analyses with the CD group divided into high and low CU traits subgroups (based on the ICU) using the normative age- and sex-specific cut-offs provided by Kemp et al. (2023). The ICU cut-offs for being high in CU traits were ≥ 34 in males and ≥ 30 in females - 68 of the CD group (37% female) were classified as being low in CU traits and 96 were classified as being high in CU traits (50% female). While this does not correspond exactly to the Limited Prosocial Emotions specifier for CD in the DSM-5 (Colins et al., 2021), it is a good proxy as many of the behaviours captured in the LPE specifier (e.g., lack of concern about school performance or deficits in guilt and empathy) are assessed by the ICU. It is also advantageous to use the normative cut-offs because this means that other research groups are more likely to be able to emulate our approach and replicate our findings (Kemp et al., 2023). Finally, due to evidence indicating that brain development may follow a non-linear trajectory in certain brain regions, we repeated the analyses including a quadratic age term (age^2^). By incorporating quadratic models, we aimed to identify developmental peaks that cannot be adequately estimated using linear models alone (Shaw et al., 2007).

## Results

Table 1 reports the sample’s demographic and clinical characteristics. The CD and HC groups did not significantly differ in age, pubertal stage and handedness. However, female participants were significantly older and had higher pubertal development scores than male participants. Additionally, a significant interaction between sex and group was found for handedness, where males with CD were more likely to be left-handed than male HCs, while the opposite pattern was observed in females. As expected, the male and female CD groups had significantly more CD symptoms and higher levels of CU traits, but lower IQs than male and female HCs. Males with CD had higher rates of comorbid ADHD than their female counterparts. Unsurprisingly, the CD group had significantly higher rates of comorbid lifetime diagnoses of ODD, depression and anxiety disorders than the control group as the presence of psychiatric conditions was an exclusion criterion for the HCs. Furthermore, females with CD were significantly more likely to comorbid depression or anxiety disorders than their male counterparts.

### Imaging Findings: Main Effects of Group

**Fig. 1:**
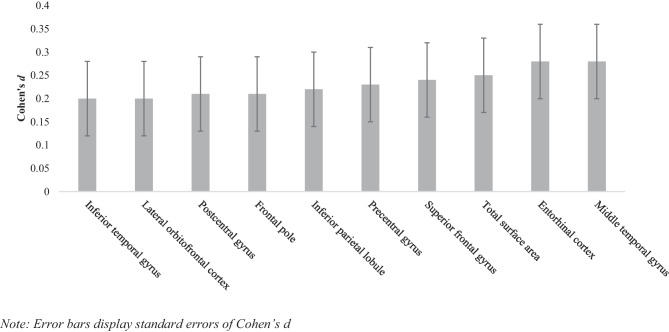
Effect sizes of the group differences (CD < HC) in cortical surface area

Relative to controls, the CD group had lower surface area across multiple frontal, temporal and parietal regions (see Table 2; Fig. S2 in the Supplement displays the group means). Effect sizes (reported as Cohen’s* d*) in these regions were small and ranged from 0.20-0.28 (see Fig. 1) with the largest effects observed in the middle temporal gyrus and entorhinal cortex. No main effects of group were observed for cortical thickness (CT) or subcortical volume following correction for multiple comparisons. The complete results of the group comparisons for CT, SA and subcortical volumes are reported in Tables S4–S6 (see Supplement). Furthermore, no significant two-way interactions between group and age, or three-way interactions between group, age and sex were found after controlling for multiple comparisons.

**Table 2:** Main effects of group (CD < HC) for cortical surface area

**Region**	**CD (N)**	**HC (N)**	**Cohen’s* d***	**SE**	**95% CIs**	***p***	***P***_***FDR***_	**Sensitivity** **IQ/ ADHD/ Anx/ Dep**
**Entorhinal cortex**	358	279	0.28	0.08	0.12, 0.43	0.001	0.011	Yes/ Yes/ Yes/ Yes
**Middle temporal gyrus**	363	278	0.28	0.08	0.12, 0.43	0.001	0.011	Yes/ Yes/ Yes/ Yes
**Total surface area**	379	291	0.25	0.08	0.10, 0.40	0.002	0.019	No/ Yes/ Yes/ Yes
**Superior frontal gyrus**	370	288	0.24	0.08	0.09, 0.39	0.002	0.021	Yes/ No/ Yes/ Yes
**Precentral gyrus**	350	270	0.23	0.08	0.07, 0.38	0.006	0.035	No/ No/ Yes/ Yes
**Inferior parietal lobule**	367	283	0.22	0.08	0.07, 0.38	0.006	0.035	Yes/ Yes/ Yes/ Yes
**Postcentral gyrus**	341	266	0.21	0.08	0.05, 0.37	0.010	0.043	No/ Yes/ Yes/ Yes
**Frontal pole**	373	290	0.21	0.08	0.06, 0.36	0.008	0.042	No/ Yes/ Yes/ Yes
**Inferior temporal gyrus**	360	281	0.20	0.08	0.04, 0.36	0.013	0.045	No/ Yes/ Yes/ Yes
**Lateral orbitofrontal cortex**	377	290	0.20	0.08	0.05, 0.35	0.011	0.043	No/ No/ Yes/ Yes

The table displays group differences that survive when applying a False Discovery Rate correction for multiple comparisons at q=0.05. Results of the sensitivity analyses are indicated under ‘Sensitivity’, Yes = significant prior to FDR correction, No = not significant prior to FDR correction, for results following FDR correction see Table S4 in Supplement. Regions are the average of left and right hemisphere surface area. The model is adjusted for sex, age, intracranial volume and site and included all possible two-way and three-way interactions between group, sex and age

*FDR* false discovery rate, *CD* Conduct Disorder, *HC* healthy controls, *SE* standard error of Cohen’s d, *CI* confidence interval, *Sensitivity* sensitivity analyses, *Anx* Anxiety, *Dep* Depression

### Imaging Findings: Main Effects of Age

Our analyses further showed that cortical thickness declines within the age bracket under investigation as we observed whole-brain corrected age effects in almost every cortical region (except the entorhinal cortex and temporal pole). Similarly, surface area and subcortical volume also demonstrated regional age-related declines (that were significant in 40% of cortical and 50% of subcortical regions, respectively). Main effects of age on CT, SA and subcortical volumes are reported in Tables S7–S9 (see Supplement).

Further analyses were conducted to test for potential non-linear trajectories of the examined brain areas. There was no evidence for significant main effects of age^2^ after multiple comparison correction (albeit nominally significant effects were observed in the amygdala and the thalamus). No significant group-by-age^2^ interactions were detected.

### Sensitivity Analyses

When adjusting for IQ, there were significant effects of group on surface area in the entorhinal cortex, inferior parietal lobule, as well as the middle temporal and superior frontal gyri (CD < HC). However, none of these results survived correction for multiple comparisons (see Table S10 in Supplement).

After controlling for lifetime ADHD diagnoses, group differences in SA remained significant across the entorhinal cortex, the inferior parietal lobule and temporal cortex, the middle temporal and postcentral gyrus as well as the frontal pole. However, only the finding in the middle temporal gyrus remained significant after controlling for multiple comparisons. Notably, the sample in this analysis was smaller due to missing information on ADHD diagnoses (up to 14% of data were missing for this variable) (see Table S11 in Supplement).

Group differences in SA remained significant when we controlled for anxiety disorder and depression comorbidity in separate analyses and most effects remained significant or marginally significant after correcting for multiple comparisons (see Tables S12 & S13 in the Supplement).

To understand the possible impact of CU traits on our findings, we divided the CD group into low and high CU traits subgroups, comparing them separately to the HCs. While our analysis revealed a few significant group differences for SA and group-by-age interactions in the high CU traits vs. HC comparison that were not observed in the low CU traits vs. HC comparison, and vice versa, differences were overall limited, and none survived correction for multiple comparisons. Overall, these subgroup analyses generally showed fewer significant effects than the main analyses (likely due to the reduced group sizes).

## Discussion

The aim of this study was to investigate whether brain maturation is altered in youth with CD in comparison to healthy controls, using cross-sectional MRI data from a large sample spanning the developmental period from 9 to 18 years of age. Overall, the CD group showed lower surface area in a range of cortical regions including the frontal, temporal and parietal lobes (9 out of the 34 regions tested) as well as total surface area. The largest group differences were observed in the middle temporal and entorhinal cortex (albeit with relatively small effect sizes of *d* = 0.28). These results provide evidence that CD is associated with lower SA across a range of cortical regions. Notably, in addition to these main effects of group, we were also able to replicate the main effects of age that have been observed in previous normative studies (e.g., Raznahan et al., 2011) - CT values declined with age across the majority of brain regions studied, while SA and subcortical volume decreased in 40–50% of the investigated brain areas. The lack of significant interactions between group and age suggest that this downward trajectory did not differ between youth with CD and controls.

Although we hypothesized that brain maturation might be delayed in youth with CD, no significant interactions between group and age emerged that would have supported this prediction. This runs counter to previous studies that provided some evidence for altered neurodevelopmental trajectories related to conduct problems (Albaugh et al., 2023; De Brito et al., 2009; Oostermeijer et al., 2016). Notably, Albaugh and colleagues (2023) identified significant conduct problems-by-age interactions which indicated that conduct problems were associated with cortical thinning in the oldest age groups in their sample (18+ years). This may suggest that the lack of significant group-by-age interactions (for CT) in the present study might relate to the fact that we only included participants up to age 18 and that there were comparatively fewer ‘older’ participants. Furthermore, the brain undergoes extensive development before the age of 9, with cortical thickness peaking at around age 2 and surface area peaking at age 11 (Bethlehem et al., 2022), meaning if we had included data from participants both younger than 9 and older than 18, we may have found evidence for group-by-age interactions.

Alternatively, the lack of significant group-by-age interactions might also be due to Type II error, meaning that true, but small, effects are present but were not detected by the analyses. Recent research indicates that large sample sizes may be required to detect the small neuroanatomical alterations that we might expect in psychopathologies (Marek et al., 2022) and even larger samples are likely to be needed to perform well-powered interaction analyses. Therefore, while this study included a larger sample than many previous studies, our statistical power may still not have been sufficient to detect the small interaction effects that we might expect.

The current study represents the first large-scale effort focusing on brain development in youth with diagnosed CD, as opposed to conducting a dimensional analysis with smaller numbers of children exhibiting elevated conduct problems (e.g., Albaugh et al., 2023; Oostermeijer et al., 2016). Nonetheless, these cross-sectional findings should be followed up with longitudinal analyses using larger sample sizes and including younger as well as older participants. Future research could also seek to disentangle the effects of CD and ODD symptoms on brain structure and development (Hawes et al., 2023). It was not possible to investigate this issue here because most of the participants with CD had comorbid ODD (72% of the boys and 77% of the girls).

### Cortical Surface Area

Despite the absence of significant group-by-age interactions, we identified lower total SA as well as lower SA in a number of cortical regions in youth with CD compared to HCs. The brain regions identified in the current study align with previous studies that have reported consistent evidence of alterations in frontal, temporal and parietal regions (Fairchild et al., 2015; Jiang et al., 2015; Smaragdi et al., 2017). These regions have been associated with processes such as cognitive control, emotion processing, motor control, memory and executive functioning (Blair et al., 2018; Stuss, 2011). Furthermore, the temporal lobes have previously been linked to antisocial behaviour (Hoptman, 2003), and damage to the frontal lobes has been associated with impaired social behaviour and moral reasoning (Anderson et al., 1999), which are frequently observed in youth with CD (Pardini & Fite, 2010).

Surprisingly, group differences were only observed for SA and not for CT which deviates from much of the previous SBM literature. A possible explanation may be that studies investigating SBM metrics have more often considered CT than SA (e.g., Fahim et al., 2011; Hyatt et al., 2012), which may explain the lack of evidence for SA alterations in CD. Moreover, while existing case–control studies have provided more consistent evidence for CT alterations, group differences in SA have been regularly reported; however, the findings for this metric have been more mixed (e.g., lower SA in the OFC and dorsolateral prefrontal cortex Fairchild et al., 2015; Sarkar et al., 2015]). Notably, while large-scale neuroimaging analyses by the ENIGMA-ADHD group reported lower CT in the temporal pole and fusiform gyrus, they demonstrated more widespread reductions in SA than CT in children with ADHD versus healthy controls (Hoogman et al., 2019). This might suggest that SA alterations can be detected across various cortical regions; however, as these alterations are relatively small, larger samples are required to detect them, which earlier studies on CD have not been able to achieve (e.g., the studies cited above had Ns of 24–56). Additionally, the majority of neuroimaging studies on CD employed VBM methods to investigate alterations in gray matter volume (GMV; Fairchild et al., 2011; Huebner et al., 2008; Rogers & De Brito, 2016). Despite the distinction between GMV, SA and CT, GMV appears to be more strongly correlated with SA than CT (Winkler et al., 2012). Therefore, previous findings of alterations in GMV may have been driven by changes in SA rather than CT.

### Sensitivity Analyses

The sensitivity analyses revealed that several of the group differences in SA between the CD group and HCs remained significant when controlling for IQ or ADHD comorbidity. While most differences did not survive multiple comparison correction in these sensitivity analyses, the effect sizes remained similar to those observed in the main analysis (Cohen’s ds ~ 0.20). Additionally, the substantial rate of missing ADHD data across groups (up to 14%) is likely to have affected the statistical power of the corresponding sensitivity analysis. While it is important to control for potential confounding effects, it should be highlighted that CD is strongly associated with low IQ and ADHD diagnoses (Anney et al., 2008; Deater-Deckard et al., 2009). By isolating and controlling for these aspects, we may inadvertently have created an artificial representation that does not accurately reflect the complex reality of CD and its interplay with cognitive abilities and comorbid conditions. Thus, while it is crucial to consider and control for confounding factors, it is equally important to recognize the multifaceted nature of CD and its interactions with various cognitive and psychiatric factors when interpreting research findings.

In contrast, many group differences remained significant when depression and anxiety disorder were controlled for. Moreover, most findings remained marginally significant following FDR correction, indictaing that internalising comorbid disorder had less impact on the main findings compared to IQ and ADHD.

Additional sensitivity analyses were conducted to examine the influence of callous-unemotional (CU) traits on the study's findings. The results revealed that the high and low CU traits subgroups differed from HCs in the same direction (albeit fewer of the differences remained significant), indicating that the main effects were not driven solely by one of the CU subgroups. There was also no clear evidence that age effects differed between youth with CD with low versus high levels of CU traits (i.e., delayed brain maturation in the high CU traits subgroup only).

The developmental neuroscience literature suggests that brain development is a process characterised by both linear and non-linear changes over time. Hence, we conducted additional analyses to explore non-linear trajectories within the investigated brain areas, but did not find convincing evidence for such trajectories (most of the changes in CT, SA or volume were linear rather than non-linear). One possible explanation for these findings is the age range of our sample. Previous research suggests (sub)cortical parameters tend to peak during the first decade of life (Bethlehem et al., 2022), meaning the age range of our participants (9 to 18 years) might not have captured the critical period of non-linear developmental changes in these brain areas – i.e., most of the participants were already on the ‘downward’ slope of their developmental trajectory for CT or SA.

### Limitations and Future Recommendations

The conclusions we can draw from the current findings need to be considered in the context of the study’s limitations. Firstly, while using an atlas-based approach is common in neuroimaging research and allows for area-specific estimations which may provide more easily interpretable results, advantages of vertex-based approaches have been established (Winkler et al., 2012). Vertex-based approaches measure cortical parameters at each vertex throughout the whole brain which allows for detection of significant group differences in regions of the brain that extend across the boundaries of established areas, increasing sensitivity to detect structural alterations (Backhausen et al., 2022).

Another important limitation of the study is the use of cross-sectional MRI data to explore developmental changes (or group differences therein). It has been argued that drawing longitudinal inferences from cross-sectional analysis should be done with caution due to issues such as interindividual differences in brain development and cohort biases (Robinson et al., 2008). Therefore, the use of longitudinal data would allow a more accurate representation of developmental trajectories (Kraemer, 2000). However, no longitudinal dataset of a similar size that contains a large number of participants with CD is currently available.

Additionally, it should be noted that although information on race and/or ethnicity was not collected systematically across all sites due to ethical guidelines in some of the participating countries (e.g., Germany), most (~ 90%) of the participants were born in the country of assessment and were of white European ancestry. Hence, these findings are not representative of a diverse world population and should not be interpreted as such. This also offers an opportunity for future research in exploring populations from other racial and ethnic backgrounds.

Notably, while most previous CD studies were limited by small sample sizes, the current study has greater statistical power due to the relatively large sample size. On the other hand, when considering participant numbers per age bracket, it should be noted that there were more participants in the older than the younger age brackets (see Table S2). It is also possible that we were underpowered to detect group-by-age or group-by-age-by-sex interactions. Overall, future research would therefore benefit from using longitudinal data in a similar research design (cf. Shaw et al., 2007), including younger participants and adopting a vertex-wise approach, which should enhance our ability to detect group effects and group-by-age interactions in cortical structure if they exist.

## Conclusion

This study demonstrates that youth with CD present widespread reductions in cortical surface area (especially in temporal, frontal and parietal lobes) relative to their typically developing counterparts. Structural alterations in these regions may contribute to the impairments in emotion recognition, empathy, decision-making, and moral reasoning that are characteristic of CD and its subtypes. While the study’s primary aim was to investigate brain maturation in youth with CD compared to healthy controls, no convincing evidence was found for group differences in brain development (or three-way interactions between group, age and sex). Future research should use longitudinal research designs to investigate these issues further and broaden the developmental window to consider younger children, as we may have started assessing the children too late to detect altered brain development.

### Supplementary Information

Below is the link to the electronic supplementary material.

Supplementary file1 (DOCX 180 KB)

## Data Availability

Data supporting this study are not publicly available but can be requested from the FemNAT-CD Steering Committee, which is chaired by Professor Christine M. Freitag: c.freitag@em.uni-frankfurt.de.
