# Supplementary material for: Does the Relationship between Age and Brain Structure Differ in Youth with Conduct Disorder?
Source: Res Child Adolesc Psychopathol. 2024 Apr 1;52(7):1135–46. doi: 10.1007/s10802-024-01178-w (PMC11217071; doi:10.1007/s10802-024-01178-w)
Supplement: Supplementary file 1 — Supplementary file1 (DOCX 180 KB) [file 10802_2024_1178_MOESM1_ESM.docx]

**SUPPLEMENTARY INFORMATION**

**Does the Relationship between Age and Brain Structure Differ in Youth with Conduct Disorder?**

Sarah Koerner^1^, M.Sc., Marlene Staginnus^1^, M.Res., Harriet Cornwell^1^, Ph.D., Areti Smaragdi^2^, Ph.D., Karen González-Madruga^3^, Ph.D., Ruth Pauli^4^, Ph.D., Jack C. Rogers^5^, Ph.D., Yidian Gao^4^, Ph.D., Sally Chester^4^, MSc., Sophie Townend^1^, MSc., Anka Bernhard, Ph.D.^6^, Anne Martinelli^6,7^, Ph.D., Gregor Kohls^8,9^, Ph.D., Nora Maria Raschle^11,12^, Ph.D., Kerstin Konrad^8,13^, Ph.D., Christina Stadler^10^, Ph.D., Christine M. Freitag^8^, M.D., Ph.D. (Habilitation), Stephane A. De Brito^4^, Ph.D., and Graeme Fairchild^1^, Ph.D.

Affiliations:

^1^ Department of Psychology, University of Bath, Bath, UK

^2^ Child Development Institute, Toronto, Canada

^3^ Department of Psychology, Middlesex University, London, UK

^4^ Centre for Human Brain Health, School of Psychology, University of Birmingham, Birmingham, UK

^5^ Institute for Mental Health, School of Psychology, University of Birmingham, Birmingham, UK

^6^ Department of Child and Adolescent Psychiatry, Psychosomatics and Psychotherapy, University Hospital Frankfurt, Goethe University, Frankfurt am Main, Germany

^7^ School of Psychology, Fresenius University of Applied Sciences, Frankfurt, Germany

^8^ Child Neuropsychology Section, Department of Child and Adolescent Psychiatry, Psychosomatics and Psychotherapy, University Hospital, RWTH Aachen, Aachen, Germany

^9^ Department of Child and Adolescent Psychiatry, Medical Faculty, TU Dresden, Dresden, Germany

^10^ Department of Child and Adolescent Psychiatry, Psychiatric University Hospital, University of Basel, Basel, Switzerland

^11^ Jacobs Center for Productive Youth Development at the University of Zurich, Zurich, Switzerland

^12^ Neuroscience Centre Zurich (ZNZ), University and ETH Zurich, Zurich, Switzerland

^13^ JARA- Brain Institute II, Molecular Neuroscience and Neuroimaging, RWTH Aachen and Research Centre Juelich, Juelich, Germany

**Corresponding Author:**

Sarah Koerner, [sskoerner@gmail.com](mailto:sskoerner@gmail.com)

**Supplementary Methods**

**Fig. S1**

*Flowchart of the participant inclusion process*

Full FemNAT-CD sample

N = 1743

Excluded: n = 87

**3**: Excluded by consortium (e.g., exclusion criterion fulfilled)

**1**: Duplicate scan

**5**: No raw T1 data available

**69**: Excluded prior to QC due to poor T1 quality

**9**: Failed ENIGMA Quality control process

Structural MRI data available

N = 769

Structural MRI data available post ENIGMA QC

N = 682

Participants excluded due to missing demographic data

N = 12

Sample included in analyses

N = 670 (CD = 291; HC = 379)

MRI=Magnetic Resonance Imaging; CD=Conduct Disorder; HC=Healthy controls; QC=Quality Control

**Table S1.**

*Number of participants per contributing site.^a^*

| Group | Site 1  Frankfurt | Site 2  Aachen | Site 3 Southampton | Site 4  Basel | Site 5  Birmingham |
| --- | --- | --- | --- | --- | --- |
| CD male | 32 | 37 | 31 | 12 | 41 |
| CD female | 21 | 45 | 22 | 31 | 19 |
| HC male | 36 | 36 | 35 | 11 | 41 |
| HC female | 36 | 53 | 36 | 34 | 61 |
| Total | **125** | **171** | **124** | **88** | **162** |

^a^CD=Conduct Disorder; HC=Healthy controls

**Table S2.**

*Number of participants included in each age group, split by group and sex. ^a^*

| Age (years) | Female CD | Male CD | Female HC | Male HC |
| --- | --- | --- | --- | --- |
| 9 | 2 | 6 | 11 | 8 |
| 10 | 4 | 9 | 17 | 10 |
| 11 | 4 | 11 | 10 | 13 |
| 12 | 11 | 20 | 12 | 9 |
| 13 | 7 | 12 | 17 | 20 |
| 14 | 25 | 23 | 30 | 23 |
| 15 | 31 | 19 | 30 | 21 |
| 16 | 27 | 22 | 42 | 21 |
| 17 | 20 | 19 | 37 | 22 |
| 18 | 7 | 12 | 13 | 13 |

^a^CD=Conduct Disorder; HC=Healthy controls

**Ethical approvals**

The study was conducted in accordance with the legal regulations of the European Union, national legislations, and the Declaration of Helsinki. The original study protocols were approved by the relevant ethical committees at each site prior to the start of data collection: the Ethics Committee of the Medical Faculty of Goethe University Frankfurt for the Frankfurt site (site 1), RWTH Aachen University Hospital (EK027/14) for the Aachen site (site 2), the Ethics Commission Northwest and Central Switzerland (EKNZ: 336/13) for the Basel site (site 4), and the Southampton University Ethics Committee (ERGO Number: 18970) and National Health Service Research Ethics Committee (NRES Committee West Midlands, Edgbaston; REC reference 13/WM/0483) for the UK sites (Southampton [site 3] and Birmingham [site 5)]). At German-speaking sites (Basel, Frankfurt, Aachen), youths aged 18 years provided informed consent, while younger participants provided assent and their parent/caregiver informed consent. At the UK sites (Southampton, Birmingham), informed consent was obtained from youths aged 16 years or over, whereas those younger than this provided assent and informed consent was obtained from their parents or caregivers. Ethical approval for the current analyses was obtained from the University of Bath Psychology Research Ethics Committee (Ethics reference: 19-297).

**Table S3.**

*Scanning parameters and acquisition sequences at each site.*

|  | Site 1  Frankfurt | Site 2  Aachen | Site 3  Southampton | Site 4  Basel | Site 5  Birmingham |
| --- | --- | --- | --- | --- | --- |
| Scanner make and model | Siemens  Magnetom Tim Trio | Siemens  Magnetom Prisma | Siemens Magnetom Tim Trio | Siemens  Magnetom Prisma | Philips Achieva |
| Software version | Syngo MR A35 | Syngo MR D13D | Syngo MR B17 | Syngo MR D13D | Version 3.2.6.1 |
| Head coil | 8 channel | 20 channel | 32 channel | 20 channel | 32 channel |
| T1-weighted  MPRAGE  scanning parameters | TE = 3.4 ms,  TR = 1900 ms,  flip angle = 9°,  FHxAP field of view (FoV) = 256 mm,  RL FoV = 192 mm, matrix = 256,  voxel size = 1x1x1 mm,  sagittal slices = 192,  bandwidth = 180 Hz/pixels,  total scan time = 4 minutes 26 seconds | TE = 3.4 ms,  TR = 1900 ms,  flip angle = 9°,  FHxAP field of view (FoV) = 256 mm,  RL FoV = 192 mm, matrix = 256,  voxel size = 1x1x1 mm,  sagittal slices = 192,  bandwidth = 180 Hz/pixels,  total scan time = 4 minutes 26 seconds | TE = 3.4 ms,  TR = 1900 ms,  flip angle = 9°,  FHxAP field of view (FoV) = 256 mm,  RL FoV = 192 mm, matrix = 256,  voxel size = 1x1x1 mm,  sagittal slices = 192,  bandwidth = 180 Hz/pixels,  total scan time = 4 minutes 26 seconds | TE = 3.4 ms,  TR = 1900 ms,  flip angle = 9°,  FHxAP field of view (FoV) = 256 mm,  RL FoV = 192 mm, matrix = 256,  voxel size = 1x1x1 mm,  sagittal slices = 192,  bandwidth = 180 Hz/pixels,  total scan time = 4 minutes 26 seconds | TE = 3.7 ms,  TR = 1900 ms,  flip angle = 9°,  FHxAP field of view (FoV) = 256 mm,  RL FoV = 192 mm, matrix = 256,  voxel size = 1x1x1 mm,  sagittal slices = 192,  bandwidth = 174 Hz/pixels,  total scan time = 6 minutes 5 seconds |

**Site qualification procedures for MRI data acquisition**

To ensure comparability of MRI data across the five scanning sites, each site adopted similar scanning parameters and image acquisition sequences (see Table S3) and underwent site qualification procedures to ensure that sequences were comparable. These included scanning an American College of Radiology (ACR) phantom (Chen et al., 2004), a Functional Biomedical Informatics Research Network (FBIRN) phantom (Glover, 2005), and a human volunteer. The ACR phantom is designed to assess structural MRI sequences, and the FBIRN is designed to assess scanner stability during functional MRI sequences, and provide information concerning scanner drift, percent fluctuation in signal, signal-to-noise ratio, and signal-to-fluctuation-noise ratio. Once collected, the datasets were reviewed by an MRI physicist at the University of Birmingham (Dr. Ali Chowdhury), and each site adjusted the scanning parameters according to the physicist’s recommendations until the sites’ scanning procedures were comparable. The sites were only able to start collecting data once they had successfully passed this site qualification procedure step.

**Supplementary Results**

**Fig. S2**

*Group differences in cortical surface area (mm^2^)*

2A 2B

2C 2D

2E 2F

2G 2H

2I 2J

*Bars display the adjusted means of cortical surface area. Error bars display the standard error of the adjusted means. P-values display the FDR corrected p-values. CD = Conduct Disorder. HC = Healthy control*

## **Table S4.**

*Group Comparisons for Cortical Thickness.^a^*

| Region | CD (N) | HC (N) | Cohen’s d | SE d | 95% CIs | *p* | *P*_FDR_ |
| --- | --- | --- | --- | --- | --- | --- | --- |
| Banks superior temporal sulcus | 285 | 375 | 0.18 | 0.08 | 0.03, 0.34 | 0.022 | 0.766 |
| Caudal anterior cingulate cortex | 286 | 373 | -0.14 | 0.08 | -0.03, 0.01 | 0.077 | 0.844 |
| Caudal middle frontal gyrus | 280 | 373 | -0.07 | 0.08 | -0.23, 0.08 | 0.358 | 0.964 |
| Cuneus | 285 | 367 | -0.09 | 0.08 | -0.24, 0.06 | 0.263 | 0.844 |
| Entorhinal cortex | 279 | 358 | -0.11 | 0.08 | -0.26,0.05 | 0.180 | 0.844 |
| Frontal pole | 290 | 373 | -0.05 | 0.08 | -0.20, 0.10 | 0.525 | 0.979 |
| Fusiform gyrus | 288 | 376 | 0.00 | 0.08 | -0.15, 0.15 | 0.985 | 0.985 |
| Inferior parietal lobule | 283 | 367 | 0.00 | 0.08 | -0.16, 0.15 | 0.951 | 0.979 |
| Inferior temporal gyrus | 281 | 360 | -0.01 | 0.08 | -0.16, 0.15 | 0.930 | 0.979 |
| Insula | 283 | 363 | -0.04 | 0.08 | -0.20, 0.11 | 0.591 | 0.979 |
| Isthmus of the cingulate gyrus | 287 | 377 | -0.12 | 0.08 | -0.27, 0.03 | 0.129 | 0.844 |
| Lateral occipital sulcus | 284 | 368 | 0.01 | 0.08 | -0.14, 0.16 | 0.908 | 0.979 |
| Lateral orbitofrontal cortex | 290 | 377 | -0.01 | 0.08 | -0.17, 0.14 | 0.864 | 0.979 |
| Lingual gyrus | 291 | 375 | -0.06 | 0.08 | -0.21, 0.09 | 0.437 | 0.979 |
| Mean cortical thickness | 291 | 379 | 0.01 | 0.08 | -0.14, 0.17 | 0.853 | 0.979 |
| Medial orbitofrontal cortex | 287 | 372 | 0.01 | 0.08 | -0.15, 0.16 | 0.928 | 0.979 |
| Middle temporal gyrus | 278 | 363 | 0.05 | 0.08 | -0.11, 0.20 | 0.552 | 0.979 |
| Paracentral lobule | 291 | 378 | 0.09 | 0.08 | -0.06, 0.25 | 0.229 | 0.844 |
| Parahippocampal gyrus | 291 | 377 | 0.10 | 0.08 | -0.06, 0.25 | 0.227 | 0.844 |
| Pars opercularis | 291 | 378 | 0.04 | 0.08 | -0.12, 0.19 | 0.647 | 0.979 |
| Pars orbitalis | 290 | 379 | 0.05 | 0.08 | -0.11, 0.20 | 0.560 | 0.979 |
| Pars triangularis | 290 | 376 | 0.02 | 0.08 | -0.13, 0.18 | 0.759 | 0.979 |
| Pericalcarine cortex | 284 | 358 | -0.05 | 0.08 | -0.21, 0.10 | 0.532 | 0.979 |
| Postcentral gyrus | 266 | 341 | 0.15 | 0.08 | -0.01, 0.31 | 0.069 | 0.844 |
| Posterior cingulate cortex | 291 | 377 | -0.09 | 0.08 | -0.24, 0.06 | 0.265 | 0.844 |
| Precentral gyrus | 270 | 350 | 0.13 | 0.08 | -0.03, 0.29 | 0.106 | 0.844 |
| Precuneus | 289 | 377 | 0.02 | 0.08 | -0.13, 0.17 | 0.774 | 0.979 |
| Rostral anterior cingulate cortex | 288 | 375 | -0.08 | 0.08 | -0.23, 0.07 | 0.316 | 0.922 |
| Rostral middle frontal gyrus | 284 | 374 | 0.03 | 0.08 | -0.13, 0.18 | 0.741 | 0.979 |
| Superior frontal gyrus | 288 | 370 | -0.10 | 0.08 | -0.25, 0.05 | 0.212 | 0.844 |
| Superior parietal lobule | 273 | 355 | -0.05 | 0.08 | -0.21, 0.11 | 0.529 | 0.979 |
| Superior temporal gyrus | 270 | 354 | -0.03 | 0.08 | -0.19, 0.13 | 0.695 | 0.979 |
| Supramarginal gyrus | 271 | 356 | -0.02 | 0.08 | -0.18, 0.14 | 0.802 | 0.979 |
| Temporal pole | 274 | 357 | 0.03 | 0.08 | -0.13, 0.19 | 0.704 | 0.979 |
| Transverse temporal gyrus | 291 | 379 | -0.01 | 0.08 | -0.16, 0.14 | 0.910 | 0.979 |

*^a^FDR = false discovery rate. Regions are the average of left and right hemisphere surface area. The model is adjusted for sex, age, intracranial volume and site and included all two-way and three-way interactions between group, sex and age. CD = Conduct Disorder. HC = healthy controls. SE = standard error of Cohen’s d. CI = confidence interval. Positive effect sizes reflect CD < HC, while negative effect sizes indicated CD > HC.*

## **Table S5.**

*Group Comparisons for Cortical Surface Area. ^a^*

| Region | CD (N) | HC (N) | Cohen’s d | SE | 95% CIs | *p* | *P*_FDR_ |
| --- | --- | --- | --- | --- | --- | --- | --- |
| Banks superior temporal sulcus | 285 | 375 | 0.18 | 0.08 | 0.03, 0.33 | 0.022 | 0.071 |
| Caudal anterior cingulate cortex | 286 | 373 | 0.17 | 0.08 | 0.02, 0.32 | 0.032 | 0.086 |
| Caudal middle frontal gyrus | 280 | 373 | 0.16 | 0.08 | 0.00, 0.31 | 0.053 | 0.123 |
| Cuneus | 285 | 367 | 0.07 | 0.08 | -0.08, 0.23 | 0.352 | 0.456 |
| Entorhinal cortex | 279 | 358 | 0.28 | 0.08 | 0.12, 0.43 | 0.001 | 0.011 |
| Frontal pole | 290 | 373 | 0.21 | 0.08 | 0.06, 0.36 | 0.008 | 0.042 |
| Fusiform gyrus | 288 | 376 | 0.09 | 0.08 | -0.07, 0.24 | 0.282 | 0.396 |
| Inferior parietal lobule | 283 | 367 | 0.22 | 0.08 | 0.07, 0.38 | 0.006 | 0.035 |
| Inferior temporal gyrus | 281 | 360 | 0.20 | 0.08 | 0.04, 0.36 | 0.013 | 0.045 |
| Insula | 283 | 363 | 0.10 | 0.08 | -0.05, 0.26 | 0.209 | 0.332 |
| Isthmus of the cingulate gyrus | 287 | 377 | 0.12 | 0.08 | -0.03, 0.27 | 0.138 | 0.230 |
| Lateral occipital sulcus | 284 | 368 | 0.01 | 0.08 | -0.15, 0.16 | 0.927 | 0.948 |
| Lateral orbitofrontal cortex | 290 | 377 | 0.20 | 0.08 | 0.05, 0.35 | 0.011 | 0.043 |
| Lingual gyrus | 291 | 375 | 0.09 | 0.08 | -0.06, 0.24 | 0.251 | 0.381 |
| Medial orbitofrontal cortex | 287 | 372 | 0.04 | 0.08 | -0.11, 0.20 | 0.580 | 0.655 |
| Middle temporal gyrus | 278 | 363 | 0.28 | 0.08 | 0.12, 0.43 | 0.001 | 0.011 |
| Paracentral lobule | 291 | 378 | 0.06 | 0.08 | -0.09, 0.22 | 0.412 | 0.515 |
| Parahippocampal gyrus | 291 | 377 | 0.15 | 0.08 | 0.00, 0.31 | 0.050 | 0.123 |
| Pars opercularis | 291 | 378 | 0.02 | 0.08 | -0.13, 0.17 | 0.784 | 0.831 |
| Pars orbitalis | 290 | 379 | 0.18 | 0.08 | 0.03, 0.33 | 0.025 | 0.072 |
| Pars triangularis | 290 | 376 | 0.01 | 0.08 | -0.15, 0.16 | 0.948 | 0.948 |
| Pericalcarine cortex | 284 | 358 | 0.03 | 0.08 | -0.13, 0.18 | 0.742 | 0.812 |
| Postcentral gyrus | 266 | 341 | 0.21 | 0.08 | 0.05, 0.37 | 0.010 | 0.043 |
| Posterior cingulate cortex | 291 | 377 | 0.14 | 0.08 | -0.01, 0.29 | 0.073 | 0.142 |
| Precentral gyrus | 270 | 350 | 0.23 | 0.08 | 0.07, 0.38 | 0.006 | 0.035 |
| Precuneus | 289 | 377 | 0.14 | 0.08 | -0.02, 0.29 | 0.085 | 0.157 |
| Rostral anterior cingulate cortex | 288 | 375 | 0.05 | 0.08 | -0.10, 0.20 | 0.532 | 0.621 |
| Rostral middle frontal gyrus | 284 | 374 | 0.13 | 0.08 | -0.03, 0.28 | 0.116 | 0.203 |
| Superior frontal gyrus | 288 | 370 | 0.24 | 0.08 | 0.09, 0.39 | 0.002 | 0.021 |
| Superior parietal lobule | 273 | 355 | 0.09 | 0.08 | -0.07, 0.24 | 0.294 | 0.396 |
| Superior temporal gyrus | 270 | 354 | 0.15 | 0.08 | -0.01, 0.31 | 0.064 | 0.135 |
| Supramarginal gyrus | 271 | 356 | 0.15 | 0.08 | -0.01, 0.31 | 0.066 | 0.135 |
| Temporal pole | 274 | 357 | 0.09 | 0.08 | -0.07, 0.24 | 0.288 | 0.396 |
| Transverse temporal gyrus | 291 | 379 | 0.06 | 0.08 | -0.09, 0.21 | 0.433 | 0.522 |
| Total Surface area | 291 | 379 | 0.25 | 0.08 | 0.10, 0.40 | 0.002 | 0.019 |

*^a^FDR = false discovery rate. Regions are the average of left and right hemisphere surface area. The model is adjusted for sex, age, intracranial volume and site and included all two-way and three-way interactions between group, sex and age. CD = Conduct Disorder. HC = healthy controls. SE = standard error of Cohen’s d. CI = confidence interval. Positive effect sizes reflect CD < HC.*

**Table S6.**

*Group Comparisons for Subcortical Volumes and Intracranial Volume. ^a^*

| Region (Direction) | CD (N) | HC (N) | Cohen’s d | SE | 95% CI | *p* | *P_FDR_* |
| --- | --- | --- | --- | --- | --- | --- | --- |
| Nucleus Accumbens | 291 | 379 | 0.07 | 0.08 | -0.08, 0.23 | 0.346 | 0.396 |
| Amygdala | 289 | 377 | 0.14 | 0.08 | -0.02, 0.29 | 0.084 | 0.334 |
| Caudate | 269 | 342 | 0.09 | 0.08 | -0.07, 0.25 | 0.277 | 0.369 |
| Hippocampus | 290 | 378 | 0.21 | 0.08 | 0.05, 0.36 | 0.009 | 0.071 |
| ICV | 291 | 379 | 0.12 | 0.08 | -0.03, 0.27 | 0.135 | 0.340 |
| Pallidum | 282 | 371 | -0.11 | 0.08 | -0.26, 0.04 | 0.170 | 0.340 |
| Putamen | 368 | 279 | -0.02 | 0.08 | -0.18, 0.13 | 0.762 | 0.762 |
| Thalamus | 273 | 367 | 0.09 | 0.08 | -0.07, 0.24 | 0.270 | 0.369 |

*^a^FDR=false discovery rate. Regions are the average of the left and right hemispheres. The model was adjusted for sex, age, intracranial volume (except for the analysis with intracranial volume as the outcome) and site and included all two-way and three-way interactions between group, sex and age. CD = Conduct Disorder. HC = healthy controls. SE = standard error of Cohen’s d. CI = confidence interval. Positive effect sizes reflect CD < HC, while negative effect sizes indicated CD > HC.*

**Table S7.**

*Main Effects of Age for Cortical Thickness. ^a^*

| Region | CD (N) | HC (N) | *b* | t | *p* | *P*_FDR_ |
| --- | --- | --- | --- | --- | --- | --- |
| Banks superior temporal sulcus | 285 | 375 | -0.03 | -10.98 | <0.001 | <0.001 |
| Caudal anterior cingulate cortex | 286 | 373 | -0.03 | -8.67 | <0.001 | <0.001 |
| Caudal middle frontal gyrus | 280 | 373 | -0.02 | -9.10 | < .001 | < .001 |
| Cuneus | 285 | 367 | -0.02 | -9.62 | < .001 | < .001 |
| Entorhinal cortex | 279 | 358 | 0.00 | 0.77 | 0.444 | 0.444 |
| Frontal pole | 290 | 373 | -0.04 | -8.38 | < .001 | < .001 |
| Fusiform gyrus | 288 | 376 | -0.02 | -10.33 | < .001 | < .001 |
| Inferior parietal lobule | 283 | 367 | -0.03 | -13.55 | < .001 | < .001 |
| Inferior temporal gyrus | 281 | 360 | -0.03 | -10.43 | < .001 | < .001 |
| Insula | 283 | 363 | -0.02 | -7.94 | < .001 | < .001 |
| Isthmus of the cingulate gyrus | 287 | 377 | -0.03 | -8.66 | < .001 | < .001 |
| Lateral occipital sulcus | 284 | 368 | -0.02 | -8.10 | < .001 | < .001 |
| Lateral orbitofrontal cortex | 290 | 377 | -0.03 | -12.62 | < .001 | < .001 |
| Lingual gyrus | 291 | 375 | -0.02 | -8.75 | < .001 | < .001 |
| Mean Cortical Thickness | 291 | 379 | -0.02 | -14.01 | < .001 | < .001 |
| Medial orbitofrontal cortex | 287 | 372 | -0.03 | -9.81 | < .001 | < .001 |
| Middle temporal gyrus | 278 | 363 | -0.03 | -10.05 | < .001 | < .001 |
| Paracentral lobule | 291 | 378 | -0.02 | -9.87 | < .001 | < .001 |
| Parahippocampal gyrus | 291 | 377 | -0.01 | -3.15 | 0.002 | 0.002 |
| Pars opercularis | 291 | 378 | -0.02 | -11.27 | < .001 | < .001 |
| Pars orbitalis | 290 | 379 | -0.03 | -9.72 | < .001 | < .001 |
| Pars triangularis | 290 | 376 | -0.03 | -11.57 | < .001 | < .001 |
| Pericalcarine cortex | 284 | 358 | -0.01 | -4.35 | < .001 | < .001 |
| Postcentral gyrus | 266 | 341 | -0.02 | -6.96 | < .001 | < .001 |
| Posterior cingulate cortex | 291 | 377 | -0.03 | -11.49 | < .001 | < .001 |
| Precentral gyrus | 270 | 350 | -0.01 | -5.75 | < .001 | < .001 |
| Precuneus | 289 | 377 | -0.03 | -15.06 | < .001 | < .001 |
| Rostral anterior cingulate cortex | 288 | 375 | -0.03 | -8.10 | < .001 | < .001 |
| Rostral middle frontal gyrus | 284 | 374 | -0.03 | -14.07 | < .001 | < .001 |
| Superior frontal gyrus | 288 | 370 | -0.03 | -12.13 | < .001 | < .001 |
| Superior parietal lobule | 273 | 355 | -0.02 | -10.22 | < .001 | < .001 |
| Superior temporal gyrus | 270 | 354 | -0.02 | -7.97 | < .001 | < .001 |
| Supramarginal gyrus | 271 | 356 | -0.03 | -12.56 | < .001 | < .001 |
| Temporal pole | 274 | 357 | 0.01 | 1.14 | 0.255 | 0.262 |
| Transverse temporal gyrus | 291 | 379 | -0.02 | -6.84 | < .001 | < .001 |

*^a^FDR=false discovery rate. Regions are the average of left and right hemisphere surface area. The model is adjusted for sex, age, intracranial volume and site and included all two-way and three-way interactions between group, sex and age. CD = Conduct Disorder. HC = healthy controls. SE = standard error of Cohen’s d. CI = confidence interval.*

## **Table S8.**

*Main Effects of Age for Cortical Surface Area. ^a^*

| Region | CD (N) | HC (N) | *b* | t | *p* | *P*_FDR_ |
| --- | --- | --- | --- | --- | --- | --- |
| Banks superior temporal sulcus | 285 | 375 | -5.10 | -2.43 | 0.016 | 0.039 |
| Caudal anterior cingulate cortex | 286 | 373 | -0.69 | -0.36 | 0.716 | 0.722 |
| Caudal middle frontal gyrus | 280 | 373 | -8.10 | -1.49 | 0.137 | 0.200 |
| Cuneus | 285 | 367 | -4.63 | -1.71 | 0.088 | 0.147 |
| Entorhinal cortex | 279 | 358 | -1.28 | -1.13 | 0.260 | 0.349 |
| Frontal pole | 290 | 373 | -2.31 | -4.41 | < .001 | < .001 |
| Fusiform gyrus | 288 | 376 | -17.22 | -3.20 | 0.001 | 0.008 |
| Inferior parietal lobule | 283 | 367 | -34.19 | -3.47 | 0.001 | 0.005 |
| Inferior temporal gyrus | 281 | 360 | -15.99 | -2.60 | 0.010 | 0.028 |
| Insula | 283 | 363 | -1.23 | -0.41 | 0.686 | 0.722 |
| Isthmus of the cingulate gyrus | 287 | 377 | -1.15 | -0.52 | 0.604 | 0.682 |
| Lateral occipital sulcus | 284 | 368 | -19.48 | -2.72 | 0.007 | 0.023 |
| Lateral orbitofrontal cortex | 290 | 377 | -1.56 | -0.36 | 0.722 | 0.722 |
| Lingual gyrus | 291 | 375 | -13.12 | -2.36 | 0.018 | 0.043 |
| Medial orbitofrontal cortex | 287 | 372 | -3.66 | -1.21 | 0.226 | 0.316 |
| Middle temporal gyrus | 278 | 363 | -15.62 | -2.73 | 0.007 | 0.023 |
| Paracentral lobule | 291 | 378 | -2.70 | -1.04 | 0.301 | 0.390 |
| Parahippocampal gyrus | 291 | 377 | -3.22 | -2.27 | 0.023 | 0.051 |
| Pars opercularis | 291 | 378 | -6.11 | -1.75 | 0.080 | 0.140 |
| Pars orbitalis | 290 | 379 | -0.53 | -0.43 | 0.671 | 0.722 |
| Pars triangularis | 290 | 376 | -5.29 | -1.62 | 0.106 | 0.161 |
| Pericalcarine cortex | 284 | 358 | -3.01 | -0.87 | 0.383 | 0.463 |
| Postcentral gyrus | 266 | 341 | -28.00 | -4.46 | < .001 | < .001 |
| Posterior cingulate cortex | 291 | 377 | -6.61 | -2.80 | 0.005 | 0.023 |
| Precentral gyrus | 270 | 350 | -10.76 | -1.67 | 0.096 | 0.153 |
| Precuneus | 289 | 377 | -19.94 | -3.19 | 0.001 | 0.008 |
| Rostral anterior cingulate cortex | 288 | 375 | 0.99 | 0.53 | 0.598 | 0.682 |
| Rostral middle frontal gyrus | 284 | 374 | -25.60 | -2.65 | 0.008 | 0.026 |
| Superior frontal gyrus | 288 | 370 | -22.29 | -2.16 | 0.031 | 0.064 |
| Superior parietal lobule | 273 | 355 | -50.33 | -5.64 | < .001 | < .001 |
| Superior temporal gyrus | 270 | 354 | -18.50 | -3.15 | 0.002 | 0.008 |
| Supramarginal gyrus | 271 | 356 | -17.51 | -2.50 | 0.013 | 0.034 |
| Temporal pole | 274 | 357 | -0.86 | -0.98 | 0.326 | 0.407 |
| Transverse temporal gyrus | 291 | 379 | -1.82 | -0.41 | 0.044 | 0.081 |
| Total Surface Area | 291 | 379 | -265.59 | -2.05 | 0.041 | 0.079 |

*^a^FDR=false discovery rate. Regions are the average of left and right hemisphere surface area. The model is adjusted for sex, age, intracranial volume and site and included all two-way and three-way interactions between group, sex and age. CD = Conduct Disorder. HC = healthy controls. SE = Standard error of Cohen’s d. CI = confidence interval.*

**Table S9.**

*Main Effects of Age on Subcortical Volumes and Intracranial Volume. ^a^*

| Region (Direction) | CD (N) | HC (N) | *b* | t | *p* | *P_FDR_* |
| --- | --- | --- | --- | --- | --- | --- |
| Nucleus Accumbens | 291 | 379 | -7.67 | -5.38 | < .001 | < .001 |
| Amygdala | 289 | 377 | -0.85 | -0.32 | 0.752 | 0.752 |
| Caudate | 269 | 342 | -26.33 | -3.35 | 0.001 | 0.002 |
| Hippocampus | 290 | 378 | -4.05 | -0.71 | 0.476 | 0.543 |
| ICV | 291 | 379 | 2950.97 | 1.11 | 0.266 | 0.355 |
| Pallidum | 282 | 371 | -8.75 | -2.51 | 0.012 | 0.025 |
| Putamen | 279 | 368 | -58.88 | -6.23 | < .001 | < .001 |
| Thalamus | 273 | 367 | 25.91 | 2.02 | 0.044 | 0.071 |

*^a^FDR=false discovery rate. Regions are the average of left and right hemisphere surface area. The model is adjusted for sex, age, intracranial volume (except for the analysis with intracranial volume as the outcome) and site and included all two-way and three-way interactions between group, sex and age. CD = Conduct Disorder. HC = healthy controls. SE = standard error of Cohen’s d. CI = confidence interval.*

**Table S10.**

*Group Comparisons for Cortical Surface Area when controlling for IQ. ^a^*

| Region | CD (N) | HC(N) | Cohen’s d | SE | 95% Cis | *p* | *P_FDR_* |
| --- | --- | --- | --- | --- | --- | --- | --- |
| Middle temporal gyrus | 361 | 277 | 0.21 | 0.08 | 0.05, 0.36 | 0.011 | 0.190 |
| Entorhinal cortex | 356 | 278 | 0.19 | 0.08 | 0.03, 0.34 | 0.020 | 0.190 |
| Inferior parietal lobule | 365 | 283 | 0.19 | 0.08 | 0.04, 0.35 | 0.017 | 0.190 |
| Superior frontal gyrus | 368 | 287 | 0.17 | 0.08 | 0.02, 0.33 | 0.028 | 0.190 |

*^a^FDR=false discovery rate. Regions are the average of left and right hemisphere surface area. The model is adjusted for sex, age, intracranial volume and site and included all two-way and three-way interactions between group, sex and age. Only regions that showed significant group differences prior to multiple comparison adjustment are displayed. CD = Conduct Disorder. HC = healthy controls. SE = standard error of Cohen’s d. CI = confidence interval. Positive effect sizes reflect CD < HC.*

**Table S11.**

*Group Comparisons for Cortical Surface Area when controlling for ADHD. ^a^*

| Region | CD (N) | HC(N) | Cohen’s d | SE | 95% Cis | *p* | *P_FDR_* |
| --- | --- | --- | --- | --- | --- | --- | --- |
| Middle temporal gyrus | 345 | 242 | 0.29 | 0.08 | 0.13, 0.45 | 0.001 | 0.035 |
| Entorhinal cortex | 340 | 242 | 0.25 | 0.08 | 0.08, 0.41 | 0.004 | 0.070 |
| Supramarginal gyrus | 340 | 239 | 0.23 | 0.08 | 0.06, 0.39 | 0.009 | 0.088 |
| Inferior temporal gyrus | 343 | 243 | 0.22 | 0.08 | 0.06, 0.38 | 0.010 | 0.088 |
| Banks superior temporal sulcus | 358 | 248 | 0.20 | 0.08 | 0.04, 0.36 | 0.019 | 0.105 |
| Frontal Pole | 355 | 252 | 0.20 | 0.08 | 0.04, 0.36 | 0.016 | 0.105 |
| Postcentral gyrus | 325 | 238 | 0.19 | 0.08 | 0.03, 0.36 | 0.027 | 0.105 |
| Caudal anterior cingulate cortex | 355 | 249 | 0.18 | 0.08 | 0.02, 0.34 | 0.029 | 0.105 |
| Inferior parietal lobule | 351 | 248 | 0.18 | 0.08 | 0.02, 0.34 | 0.030 | 0.105 |
| Surface Area | 361 | 253 | 0.18 | 0.08 | 0.02, 0.34 | 0.027 | 0.105 |
| Precuneus | 359 | 251 | 0.17 | 0.08 | 0.01, 0.33 | 0.041 | 0.130 |

*^a^FDR=false discovery rate. Regions are the average of left and right hemisphere surface area. The model is adjusted for sex, age, intracranial volume and site and included all two-way and three-way interactions between group, sex and age. Only regions that showed significant group differences prior to multiple comparison adjustment are displayed.CD = Conduct Disorder. HC = healthy controls. SE = standard error of Cohen’s d. CI = confidence interval. 56 participants (8%) were missing data on ADHD diagnoses. Positive effect sizes reflect CD < HC.*

**Table S12.**

*Group Comparisons for Cortical Surface Area when controlling for Anxiety Disorders. ^a^*

| Region | CD (N) | HC(N) | Cohen’s d | SE | 95% Cis | *p* | *P_FDR_* |
| --- | --- | --- | --- | --- | --- | --- | --- |
| Entorhinal cortex | 344 | 273 | 0.29 | 0.08 | 0.13, 0.45 | 0.001 | 0.018 |
| Middle temporal gyrus | 349 | 272 | 0.25 | 0.08 | 0.09, 0.40 | 0.003 | 0.048 |
| Total Surface Area | 365 | 285 | 0.22 | 0.08 | 0.07, 0.38 | 0.006 | 0.056 |
| Inferior parietal lobule | 355 | 277 | 0.22 | 0.08 | 0.06, 0.37 | 0.008 | 0.056 |
| Superior frontal gyrus | 356 | 282 | 0.21 | 0.08 | 0.06, 0.37 | 0.008 | 0.056 |
| Inferior temporal gyrus | 347 | 275 | 0.21 | 0.08 | 0.05, 0.37 | 0.011 | 0.058 |
| Frontal Pole | 359 | 284 | 0.20 | 0.08 | 0.05, 0.36 | 0.012 | 0.058 |
| Lateral orbitofrontal cortex | 363 | 284 | 0.20 | 0.08 | 0.04, 0.45 | 0.014 | 0.058 |
| Pars orbitalis | 365 | 284 | 0.20 | 0.08 | 0.04, 0.35 | 0.015 | 0.058 |
| Banks superior temporal sulcus | 362 | 279 | 0.19 | 0.08 | 0.04, 0.35 | 0.018 | 0.060 |
| Caudal anterior cingulate cortex | 359 | 280 | 0.19 | 0.08 | 0.03, 0.35 | 0.019 | 0.060 |
| Postcentral gyrus | 329 | 261 | 0.19 | 0.08 | 0.03, 0.36 | 0.021 | 0.061 |
| Precentral gyrus | 337 | 264 | 0.16 | 0.08 | 0.00, 0.32 | 0.049 | 0.132 |

*^a^FDR=false discovery rate. Regions are the average of left and right hemisphere surface area. The model is adjusted for sex, age, intracranial volume and site and included all two-way and three-way interactions between group, sex and age. Only regions that showed significant group differences prior to multiple comparison adjustment are displayed.CD = Conduct Disorder. HC = healthy controls. SE = standard error of Cohen’s d. CI = confidence interval. 20 participants (3%) were missing data on Anxiety diagnoses. Positive effect sizes reflect CD < HC.*

**Table S13.**

*Group Comparisons for Cortical Surface Area when controlling for Depression. ^a^*

| Region | CD (N) | HC(N) | Cohen’s d | SE | 95% Cis | *p* | *P_FDR_* |
| --- | --- | --- | --- | --- | --- | --- | --- |
| Middle temporal gyrus | 349 | 272 | 0.23 | 0.08 | 0.08, 0.39 | 0.004 | 0.052 |
| Total Surface Area | 365 | 285 | 0.21 | 0.08 | 0.06, 0.37 | 0.008 | 0.052 |
| Superior frontal gyrus | 356 | 282 | 0.21 | 0.08 | 0.05, 0.37 | 0.009 | 0.052 |
| Precentral gyrus | 337 | 264 | 0.21 | 0.08 | 0.05, 0.38 | 0.010 | 0.052 |
| Inferior parietal lobule | 355 | 277 | 0.21 | 0.08 | 0.05, 0.36 | 0.010 | 0.052 |
| Inferior temporal gyrus | 347 | 275 | 0.21 | 0.08 | 0.05, 0.37 | 0.011 | 0.052 |
| Frontal Pole | 359 | 284 | 0.20 | 0.08 | 0.05, 0.36 | 0.011 | 0.052 |
| Entorhinal cortex | 344 | 273 | 0.21 | 0.08 | 0.05, 0.37 | 0.012 | 0.052 |
| Lateral orbitofrontal cortex | 363 | 284 | 0.19 | 0.08 | 0.04, 0.35 | 0.016 | 0.062 |
| Caudal anterior cingulate cortex | 359 | 280 | 0.18 | 0.08 | 0.03, 0.34 | 0.024 | 0.083 |
| Caudal middle frontal gyrus | 360 | 275 | 0.17 | 0.08 | 0.02, 0.33 | 0.031 | 0.088 |
| Postcentral gyrus | 329 | 261 | 0.18 | 0.08 | 0.02, 0.34 | 0.032 | 0.088 |
| Banks superior temporal sulcus | 362 | 279 | 0.17 | 0.08 | 0.02, 0.33 | 0.034 | 0.088 |
| Pars orbitalis | 365 | 284 | 0.17 | 0.08 | 0.01, 0.32 | 0.035 | 0.088 |

*^a^FDR=false discovery rate. Regions are the average of left and right hemisphere surface area. The model is adjusted for sex, age, intracranial volume and site and included all two-way and three-way interactions between group, sex and age. Only regions that showed significant group differences prior to multiple comparison adjustment are displayed.CD = Conduct Disorder. HC = healthy controls. SE = standard error of Cohen’s d. CI = confidence interval. 20 participants (3%) were missing data on Depression diagnoses. Positive effect sizes reflect CD < HC.*

**References**

Chen, C., Wan, Y., Wai, Y. & Liu, H. (2004). Quality assurance of clinical MRI scanners using ACR MRI phantom: preliminary results. *Journal of Digital Imaging, 17*, 279– 284.

Glover, G. (2005): *FBIRN Stability phantom QA procedures*. Stanford University and FBIRN.
